# Anal fistulas: do classification systems predict surgical outcomes?

**DOI:** 10.1590/0102-67202025000058e1927

**Published:** 2026-06-12

**Authors:** José Américo Bacchi HORA, Lucas Faraco SOBRADO, Carlos Walter SOBRADO, Natally HORVAT, Carlos Frederico Sparapan MARQUES, Ulysses RIBEIRO, Sérgio Carlos NAHAS

**Affiliations:** 1 Universidade de São Paulo, Faculty of Medicine, Department of Gastroenterology, Division of Coloproctology – São Paulo (SP), Brazil.; 2 Mayo Clinic, Department of Radiology – Rochester (MN), USA.

**Keywords:** Rectal Fistula, Anal Canal, Classification, Fecal Incontinence, Colorectal Surgery, Fístula Retal, Canal Anal, Classificação, Incontinência Fecal, Cirurgia Colorretal

## Abstract

**Background:**

Anal fistulas remain challenging in colorectal surgery, with recurrence and postoperative incontinence common despite advances in treatment. Multiple classification systems exist, but their predictive value for surgical and functional outcomes is unclear.

**Aims:**

The aim of this study was to compare Parks, American Society of Colon and Rectal Surgeons (ASCRS), and St. James’s University Hospital (SJUH) MRI-based classifications in predicting surgical outcomes, including continence preservation.

**Methods:**

Retrospective analysis of 89 patients undergoing definitive surgical treatment for anorectal fistulas at a single referral center (2012–2019). Exclusions included rectovaginal fistulas, Crohn’s disease, or prior pelvic radiotherapy. Fistulas were classified using Parks, ASCRS, and, when available, SJUH (n=49). Outcomes included the number of procedures, type of initial procedure, fistula closure, and closure without continence deterioration. Continence was assessed using the Cleveland Clinic Jorge-Wexner score.

**Results:**

Most fistulas were transsphincteric (Parks Type 2, 62%) and complex (ASCRS, 65%). Overall, 86.5% achieved fistula closure, and 73% achieved closure without continence deterioration. Parks and ASCRS were significantly associated with fistula closure with continence preservation (p=0.008 and 0.007, respectively) and type of initial procedure. Parks remained significant when considering closure alone (p=0.005), while ASCRS showed a borderline association (p=0.051). SJUH classification was associated only with procedure selection.

**Conclusions:**

Parks and ASCRS classifications were associated with fistula closure with continence preservation and type of initial procedure. Considering closure alone, only Parks remained significant. SJUH was limited to procedure selection. Overall, Parks and ASCRS guide surgical planning and prediction of functional outcomes, with Parks slightly more sensitive.

## ARTICLE HIGHLIGHTS

Parks’ classification remains the strongest predictor of treatment outcomes in anal fistula.

Parks’ and ASCRS classifications reliably predict the choice of initial surgical approach for anal fistula.

The St. James’s University Hospital MRI classification does not outperform Parks’ in predicting anal fistula treatment outcomes.

Irrespective of the surgical approach, complex anal fistulas are consistently associated with a risk of worsening fecal continence.

## Summary

**CENTRAL MESSAGE:**

Parks and ASCRS classifications are strongly associated with initial procedure choice and functional outcomes, including fistula closure with continence preservation. SJUH classification predicts only procedure selection. Parks appears slightly more sensitive, highlighting its value for surgical planning and anticipating postoperative function.

**PERSPECTIVES:**

Accurate classification of anal fistula is essential for guiding surgical strategies and counseling patient effectively. The Parks and ASCRS classification systems help predict which fistulas are likely to close successfully while preserving continence. Understanding the strengths and limitations of each system allows clinicians to select appropriate procedures, set realistic expectations, and facilitate shared decisionmaking. This approach ultimately improves outcomes in the management of complex anal fistulas.

## VISUAL ABSTRACT

**Figure:**
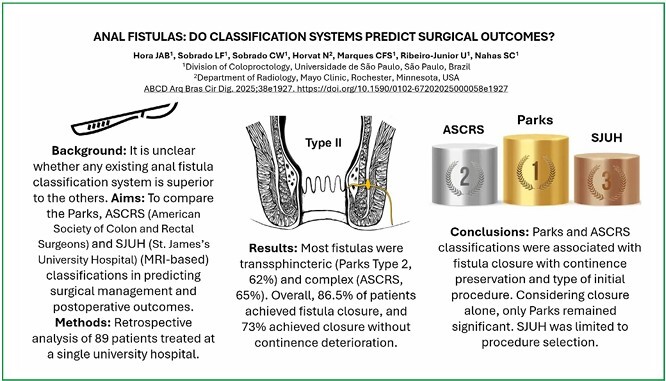

## RESUMO

**Racional:**

Fístulas anais são um desafio na cirurgia colorretal, com recorrência e incontinência pós-operatória frequentes. Diversos sistemas de classificação existem, mas seu valor preditivo para o tratamento e desfechos cirúrgicos ou funcionais permanece incerto.

**Objetivos:**

Comparar as classificações Parks, American Society of Colon and Rectal Surgeons (ASCRS) e St. James University Hospital (SJUH), baseada em ressonância magnética, quanto à predição de desfechos cirúrgicos e preservação da continência.

**Métodos:**

Análise retrospectiva de 89 pacientes submetidos a tratamento cirúrgico definitivo de fístula anal (2012–2019). Foram excluídos pacientes com fístula retovaginal, doença de Crohn ou radioterapia pélvica prévia. Fístulas foram classificadas pelos sistemas Parks, ASCRS e SJUH (n=49). Desfechos incluíram número de procedimentos, tipo de procedimento inicial, fechamento da fístula e fechamento sem piora da continência. Continência pré e pós operatória avaliada pelo escore Jorge-Wexner.

**Resultados:**

A maioria das fístulas era transesfincteriana (Parks Tipo 2, 62%) e complexa (ASCRS, 65%). O fechamento da fístula ocorreu em 86,5% e sem piora da continência em 73%. Parks e ASCRS associaram-se ao fechamento com preservação da continência (p=0,008 e p=0,007) e ao tipo de procedimento inicial. Parks manteve significância considerando apenas o fechamento (p=0,005), enquanto ASCRS apresentou associação limítrofe (p=0,051). SJUH associou-se apenas à escolha do procedimento inicial.

**Conclusões:**

Parks e ASCRS estiveram associadas ao fechamento da fístula com preservação da continência e ao tipo de procedimento inicial. Considerando apenas o fechamento, apenas Parks manteve associação significativa. SJUH esteve limitada à escolha do procedimento. No geral, Parks e ASCRS orientam planejamento cirúrgico e predição de desfechos, com Parks ligeiramente superior.

## INTRODUCTION

 Anal fistulas are a common and challenging condition in colorectal surgery, often causing pain, discharge, and impaired quality of life. Despite advances in surgical techniques, recurrence and postoperative complications, including fecal incontinence, remain frequent. Accurate characterization of fistulas is therefore essential to guide treatment and inform patients about expected outcomes. 

 Several classification systems have been proposed for anorectal fistulas, ranging from the classical anatomical frameworks of Milligan and Morgan^11^ to contemporary systems by Garg^5^ and Emile et al.^2^. While these classifications describe fistulas according to anatomy, sphincter involvement, and risk of incontinence, none has been shown to independently provide comprehensive guidance for surgical planning or reliably predict functional outcomes. 

 Among the widely used systems are the Parks classification^15^, the American Society of Colon and Rectal Surgeons (ASCRS) classification^17^, and the St. James’s University Hospital (SJUH) MRI-based classification^12^. These classifications describe fistulas according to anatomy, sphincter involvement, and the likelihood of safe fistulotomy, but their ability to predict both surgical and functional outcomes remains uncertain. 

 Preoperative assessment of fistula characteristics is essential for patient counseling, informed consent, and individualized surgical planning. Understanding which classification best correlates with postoperative healing and continence can help surgeons select the most appropriate procedure while setting realistic expectations for patients. 

 In this study, we compared the Parks, ASCRS, and SJUH classification systems to evaluate their predictive value for surgical outcomes, including treatment failure, number and type of procedures, and preservation of continence. Our goal was to identify the system that provides the most clinically useful guidance for prognosis and surgical planning in the management of anorectal fistulas. 

## METHODS

 This study is a retrospective analysis of a prospective singlecenter cohort conducted at the Hospital das Clínicas, University of São Paulo (HCFMUSP), Brazil. Patients who underwent surgical treatment for anorectal fistulas between June 13, 2012, and December 13, 2019, with at least 6 months of follow-up were included. Exclusion criteria were rectovaginal fistulas, Crohn’s disease, malignancy, anal tuberculosis, immunosuppression, or prior pelvic radiotherapy. 

**Figure 1:**
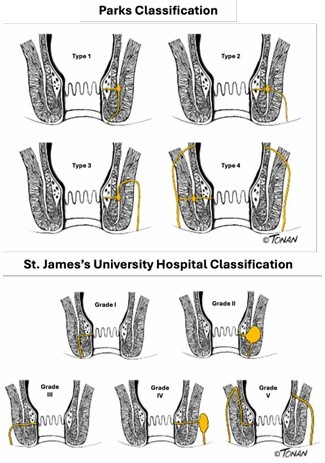
Anorectal fistula anatomy according to Parks and St. James’s University Hospital (SJUH) classification systems. Parks classification: Type 1, intersphincteric; Type 2, transsphincteric; Type 3, suprasphincteric; Type 4, extrasphincteric. SJUH classification: Grade 1, simple linear intersphincteric; Grade 2, intersphincteric with abscess or secondary tract; Grade 3, transsphincteric; Grade 4, transsphincteric with abscess or secondary tract in the ischiorectal fossa; Grade 5, supralevator or translevator extension.

 The study was approved by the Ethics Review Board of the Institution (IRB 39459320.9.0000.0068), with informed consent waived due to its retrospective design. Data were obtained from medical records and a prospective postoperative database, and MRI images were independently reviewed by two radiologists. All 89 patients were classified using the Parks and ASCRS systems, and the 49 patients who underwent MRI were additionally classified according to the SJUH system (Figure 1). Parks distinguishes four anatomical types: intersphincteric, transsphincteric, suprasphincteric, and extrasphincteric. SJUH defines Grades 1–5, from simple intersphincteric to supralevator or translevator fistulas. ASCRS categorizes fistulas as simple — amenable to single-stage fistulotomy — or complex, which includes transsphincteric fistulas involving >30% of the external sphincter, suprasphincteric or extrasphincteric tracts, horseshoe, recurrent, branching, or those associated with IBD, radiation, malignancy, preexisting incontinence, or chronic diarrhea; anterior fistulas in women are also considered complex. Classifications were applied at the time of the index surgical procedure. 

 Outcomes were analyzed in terms of the number of procedures and the type of initial procedure, categorized as examination/drainage, fistulotomy without sphincteroplasty (FWS) (simple or with cutting seton), or "other definitive procedures" (ligation of the intersphincteric tract — LIFT, rectal advancement flap, or fistulotomy with primary sphincteroplasty—FIPS). 

 Therapeutic success was defined in two ways:

1. Fistula closure; and
2. Fistula closure without deterioration of continence.

Failure was defined as recurrence, persistence, or development of a new anorectal fistula during follow-up. Continence was routinely assessed using the Cleveland Clinic Jorge-Wexner^8^ score preoperatively and at 6 months postoperatively, as part of standard clinical care. Worsening of continence was defined as new incontinence or deterioration from baseline, including progression from gas leakage to liquid or solid stool. 

 Associations between patient and fistula characteristics and outcomes were estimated using odds ratios with 95% confidence intervals via bivariate logistic regression. The number of procedures was compared across classification categories using the Mann-Whitney test for ASCRS and Kruskal-Wallis with Dunn’s post hoc test for the others. Associations between classifications and initial procedure type were assessed using chi-square or exact tests. Therapeutic outcomes were analyzed according to each classification using Fisher’s exact or likelihood ratio tests^13^. 

## RESULTS

 A total of 179 patients were initially identified, and after applying the eligibility criteria, 89 patients were included in the analysis (Table 1). Exclusions were due to Crohn’s disease (n=62), follow-up of less than 6 months (n=14), rectovaginal fistula (n=6), immunosuppression (n=5), malignancy (n=2), and tuberculosis (n=1). The cohort had a mean age of 47.4±12.6 years, 65% were male, and 69% had recurrent fistulas. Most fistulas were transsphincteric (Parks Type 2, 62%) and classified as complex by ASCRS (65%). Among the 49 patients who underwent MRI, SJUH grades were distributed as follows: Grade I, 33%; Grade IV, 31%; Grade II, 16%; Grade III, 16%; and Grade V, 4%. 

**Table 1:** Demographics, fistula classifications, and perioperative outcomes (n=89).

Variable		n (%)
Male		58 (65.2)
Age, y, mean ± SD		47.4±12.6
BMI, mean ± SD		29.1±5.3
Recurrent fistula		62 (69.7)
Preoperative fecal incontinence		14 (15.7)
Preoperative incontinence severity		
	Mild	10 (11.2)
	Moderate	3 (3.4)
	Severe	1 (1.1)
Parks classification		
	Intersphincteric	28 (31.5)
	Transsphincteric	55 (61.8)
	Suprasphincteric	6 (6.7)
	Extrasphincteric	0 (0)
ASCRS classification		
	Simple	31 (34.8)
	Complex	58 (65.2)
SJUH classification (n=49)		
	Grade I	16 (32.7)
	Grade II	8 (16.3)
	Grade III	8 (16.3)
	Grade IV	15 (30.6)
	Grade V	2 (4.1)
	Number of procedures, median (min.; max.)	1 (1; 8)
Type of initial procedure		
	Examination and drainage	24 (27)
	Fistulotomy without sphincteroplasty	39 (43.8)
	Simple fistulotomy	25
	Fistulotomy with cutting seton	14
	Other definitive procedures	26 (29.2)
	Ligation of Intersphincteric Tract	19
	Rectal Advancement Flap	5
	Fistulotomy with Primary Sphincteroplasty	2
	Fistula closure	77 (86.5)
	Fistula closure with continence preservation	65 (73)
	Continence worsening	13 (14.6)
Postoperative incontinence classification		
	Mild	17 (19.1)
	Moderate	2 (2.2)
	Failure of first definitive procedure	12 (13.5)

SD: standard deviation; BMI: body mass index; SJUH: St. James’s University Hospital; ASCRS: American Society of Colon and Rectal Surgeons.

 During a mean follow-up of 27.6±18.6 months, 150 procedures were performed. Initial procedures included FWS in 44%, other definitive procedures (LIFT, FIPS, or rectal advancement flap) in 29%, and simple examination and drainage in 27%. Overall, at the index definitive procedure, 86.5% of patients achieved fistula closure, and 73% closure without continence deterioration. New or worsened postoperative incontinence occurred in 15%, all of which was mild (Jorge-Wexner score<7), and limited to flatus in 11 of the 13 affected patients. 

 The number of procedures required varied according to fistula classification. According to Parks’ classification, intersphincteric fistulas required a median of 1 procedure (range 1–3), transsphincteric 1 (1–3), and suprasphincteric 4.5 (1–8). According to ASCRS classification, simple fistulas required 1 procedure (1–3) and complex fistulas 2 (1–8). Among patients classified by SJUH, median procedures were 1 (1–2) for Grade I, 2.5 (1–4) for Grade II, 1.5 (1–3) for Grade III, 2 (1–8) for Grade IV, and 3 (1–5) for Grade V (Table 2). 

**Table 2:** Number of procedures, initial procedure type, and fistula closure with continence preservation according to Parks, St. James’s University Hospital and St. James’s University Hospital classifications (n=89).

Classification	Type of fistula (n)	Number of procedures, median (range)	p-value	Initial procedure, n (%)			p-value	Fistula closure, n (%)		p-value	Fistula closure with continence preservation, n (%)		p-value
				Examination and drainage	Fistulotomy	Other definitive procedures		No	Yes		No	Yes	
Parks	Intersphincteric (28)	1 (1; 3)	0.001	4 (16.7)	23 (59)	1 (3.8)	<0.001	0 (0)	28 (100)	0.005	2 (7.1)	26 (92.9)	0.008
	Transsphincteric (55)	1 (1; 3)		17 (70.8)	15 (38.5)	23 (88.5)		10 (18.2)	45 (81.8)		20 (36.4)	35 (63.6)	
	Suprasphincteric (6)	4.5 (1; 8)		3 (12.5)	1 (2.6)	2 (7.7)		2 (33.3)	4 (66.7)		2 (33.3)	4 (66.7)	
ASCRS	Simple (31)	1 (1; 3)	0.004	2 (8.3)	26 (66.7)	3 (11.5)	<0.001	1 (3.2)	30 (96.8)	0.051	3 (9.7)	28 (90.3)	0.007
	Complex (58)	2 (1; 8)		22 (91.7)	13 (33.3)	23 (88.5)		11 (19)	47 (81)		21 (36.2)	37 (63.8)	
SJUH(n=49)	Grade I (16)	1 (1; 2)	0.233	3 (16.7)	8 (57.1)	5 (29.4)	0.026	2 (12.5)	14 (87.5)	0.148	3 (18.8)	13 (81.3)	0.675
	Grade II (8)	2.5 (1; 4)		3 (16.7)	2 (14.3)	3 (17.6)		0 (0)	8 (100)		3 (37.5)	5 (62.5)	
	Grade III (8)	1 (1; 3)		2 (11.1)	0 (0)	6 (35.3)		3 (37.5)	5 (62.5)		3 (37.5)	5 (62.5)	
	Grade IV (15)	2 (1; 8)		9 (50)	4 (28.6)	2 (11.8)		4 (26.7)	11 (73.3)		6 (40)	9 (60)	
	Grade V (2)	3 (1; 5)		1 (5.6)	0 (0)	1 (5.9)		1 (50)	1 (50)		1 (50)	1 (50)	

ASCRS: American Society of Colon and Rectal Surgeons; SJUH: St. James’s University Hospital.

 Patients with suprasphincteric fistulas (Parks) and complex fistulas (ASCRS) required significantly more procedures (p=0.004 and p<0.05, respectively), whereas SJUH grading was not associated with procedure count (p=0.23). Initial procedure selection varied across all classifications: suprasphincteric fistulas were more often managed with examination/drainage, intersphincteric fistulas with FWS, and transsphincteric fistulas with other definitive procedures (Parks, p<0.001). ASCRS classification also influenced initial procedure choice, with fewer complex fistulas undergoing FWS (p<0.001). SJUH grading was associated with initial procedure selection (p=0.026, p<0.05), with higher grades more often treated with examination/drainage and lower grades with FWS. 

 Analysis of therapeutic outcomes showed that both Parks and ASCRS classifications were significantly associated with fistula closure without continence deterioration (p=0.008 and p=0.007, respectively, p<0.05), whereas SJUH classification was not (p=0.675, p>0.05). When considering fistula closure alone, Parks classification remained significant (p=0.005, p<0.05), ASCRS showed a borderline association (p=0.051, p>0.05), and SJUH was not significant (p=0.148, p>0.05). Among Parks categories, continence was preserved in 93% of intersphincteric fistulas, 67% of suprasphincteric fistulas, and 64% of transsphincteric fistulas. For ASCRS classification, continence was preserved in 90% of simple and 64% of complex fistulas. 

## DISCUSSION

 In our cohort, both Parks and ASCRS classifications were significantly associated with the type of initial procedure and postoperative outcomes, including the number of procedures and fistula closure without continence deterioration. Lowerscale fistulas in both classifications achieved success rates above 90%. When considering fistula closure alone, Parks remained significant, while ASCRS showed a borderline association (p=0.051, p>0.05). In contrast, SJUH classification was not significantly associated with either fistula closure without continence deterioration or the number of procedures, likely reflecting its lower predictive value and the smaller sample size in this subgroup, as MRI was not performed in all patients. 

 The majority of patients in our series presented with complex fistulas at a ratio of approximately 2:1, consistent with the referral center nature of our institution. Transsphincteric fistulas (Parks Type 2) accounted for over half of cases, and 65% of fistulas were classified as complex according to ASCRS. This contrasts with most published series, which report a predominance of simple fistulas^1,11^. Among patients undergoing MRI (SJUH subgroup), Grades I and IV were most frequent, similar to the distribution observed by Garg in a cohort of 440 patients^5^. This likely reflects selection bias, as MRI was typically requested either to confirm anatomy in cases suspected to be suitable for simple fistulotomy (Type 1) or for more complex cases with secondary tracts (Type 4), rather than to represent the overall complexity of the cohort. 

 A trusting doctor-patient relationship and realistic prognostic counseling are essential in fistula management^14^. The number of procedures a patient may require, although rarely reported as an outcome, contributes to patient trust and can influence perceived success^18^. In our study, surgical techniques were categorized as temporary (examination/drainage) or definitive (aimed at resolving the fistula in a single intervention). Definitive procedures were further divided into FWS and "other definitive procedures," including FIPS, LIFT, and rectal advancement flap procedures. These techniques were grouped because the choice among them depends on surgeon preference and experience, although all aim to definitively treat the fistula while preserving continence in higher-risk patients. 

 In our series, both the ASCRS and Parks classifications were significantly associated with the number of procedures performed and the type of initial procedure, supporting their clinical relevance. Using the Parks system, 28 patients had intersphincteric fistulas, with only three requiring a second procedure; 55 had transsphincteric fistulas, 25 of whom required multiple procedures; and six had suprasphincteric fistulas, with only one managed in a single stage. According to ASCRS, single-stage treatment was achieved in 24 of 31 patients with simple fistulas and in 28 of 58 with complex fistulas, highlighting the system’s ability to identify low-risk cases suitable for fistulotomy. Notably, seven patients with simple fistulas did not undergo single-stage treatment due to infection, difficult tract catheterization, or intraoperative anatomical uncertainty. The SJUH classification, applied selectively to complex cases, showed no predictive value for the number of procedures. Overall, our findings align with Garg, who reported in 848 patients that lower-grade fistulas (types I–II) have a roughly 90% likelihood of successful single-stage treatment^6^. 

 Persistence and recurrence were combined into a single "failure" category. The failure rate after the first definitive procedure was 13.5%, consistent with prior literature (6–16% for simple and up to 47% for complex fistulas)^3,7,10^. When continence preservation was included as a criterion, the failure rate increased to approximately 27%, with worsening continence as the main contributor. In young men, anal fistulas and abscesses treated with fistulotomy are the primary causes of incontinence, with up to 45% experiencing some degree postoperatively^4,9,16^. Surgeons and patients may perceive success differently: surgeons often focus on anatomical closure and recurrence avoidance, while patients prioritize functional outcomes and quality of life, particularly continence. This underscores the need to incorporate both objective surgical outcomes and patient-centered functional measures when defining success. 

 Parks’ classification strongly correlated with initial procedure selection (p<0.001), emphasizing the importance of anatomy in guiding surgical strategy. ASCRS classification also influenced procedure choice: simple fistulas were treated with FWS in 83% of cases as first-line treatment, with an additional 7% receiving staged treatment, totaling 90%. Interestingly, 22% of complex fistulas were also treated with FWS, reflecting variability in surgical strategy and availability of sphincter-preserving techniques. SJUH classification was associated with initial procedure choice (p=0.026, p<0.05) but not with the number of procedures performed or postoperative outcomes. 

 Limitations of this study include its retrospective design, modest sample size, and incomplete MRI data, which limit assessment of SJUH classification relative to Parks and ASCRS systems. Strengths include a well-characterized cohort, consistent application of Parks and ASCRS classifications, and detailed follow-up with routine continence assessment using the Cleveland Clinic Jorge-Wexner score^8^, allowing evaluation of both anatomical and functional outcomes. 

## CONCLUSIONS

 Parks and ASCRS classifications were both significantly associated with the type of initial procedure and postoperative outcomes, including fistula closure with continence preservation. When considering fistula closure alone, only Parks remained significantly associated, while ASCRS showed a borderline association. SJUH classification was only associated with procedure selection and not with surgical outcomes. Overall, Parks and ASCRS provide clinically useful guidance for surgical planning and predicting functional results in anorectal fistula management, with Parks appearing slightly more sensitive in relation to surgical outcomes. 

## Data Availability

The datasets generated and/or analyzed during the current study are available from the corresponding author upon reasonable request.
